# Rational Design of the Electronic Structure of CdS Nanopowders

**DOI:** 10.1021/acs.inorgchem.3c00935

**Published:** 2023-06-29

**Authors:** Wojciech Zajac, Agnieszka Rozycka, Anita Trenczek-Zajac

**Affiliations:** † Faculty of Energy and Fuels, AGH University of Science and Technology, al. Mickiewicza 30, 30-059 Krakow, Poland; ‡ Faculty of Materials Science and Ceramics, AGH University of Science and Technology, al. Mickiewicza 30, 30-059 Krakow, Poland

## Abstract

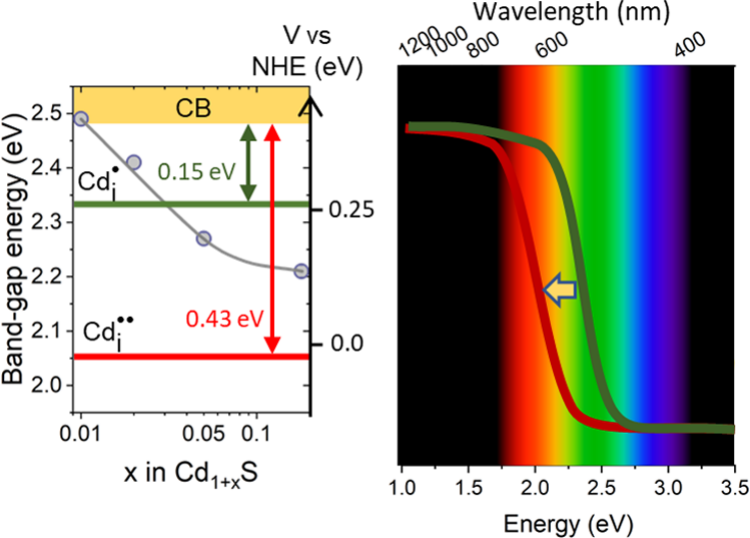

In this study, various techniques, such as energy-dispersive X-ray spectroscopy (EDX), X-ray diffraction (XRD), Raman spectroscopy, and spectrophotometry, were used to analyze the properties of nanometric CdS particles synthesized with varying precursor concentrations. EDX analysis revealed the nonstoichiometric composition manifested by an increase in the Cd/S ratio from 1.02 up to 1.43 with increasing precursor concentration. The growth of lattice parameters and unit cell volume accompanying preferential crystallization of the hexagonal phase along with an increasing Cd/S ratio was confirmed by XRD analysis. This indicated the presence of interstitial cadmium in nonstoichiometric Cd_1+*x*_S. The formation of shallow Cd_i_ donor levels below the bottom edge of the conduction band impacts the bang-gap energy; a decrease from 2.56 to 2.21 eV along with increasing nonstoichiometry was observed. This is accompanied by a widening of the range of absorption of light, which creates conditions that can lead to an increase in the efficiency of redox reactions in photochemical processes.

## Abstract 2

Precipitation of nanometric CdS led to nonstoichiometric composition manifested by cadmium-to-sulfur ratio > 1. The reason for this is the presence of a shallow donor level in the forbidden band which was confirmed by decreasing value of *E_g_*. This in turn led to the extension of the light absorption range and a potential increase in the efficiency of photochemical processes.

## 1 Introduction

Cadmium sulfide, a narrow-band-gap semiconductor, has been extensively investigated due to its unique photochemical properties. Its band gap of about 2.4 eV corresponds well with the visible range of sunlight and offers a number of potential applications. To name only the most important related to its optical properties: photocatalysts for photocatalytic water splitting and pollutants degradation, and quantum-dot-sensitized solar cells.^1−10^ Extraordinary intrinsic properties of CdS gain even more importance when moving from the micro- to the nanoscale. Nanometric cadmium sulfide has several advantages over bulk CdS.^11−13^ One advantage is that the small size of the nanoparticles increases the surface area-to-volume ratio, which can lead to increased adsorption as well as enhanced optical and electronic properties. For example, the band gap of CdS nanoparticles can be tuned by controlling their size, which can be useful for applications such as solar cells and LEDs. Another advantage is that CdS nanoparticles can be easily dispersed in a variety of solvents and matrices, which allows for easy integration into various devices and systems. Nanometric CdS has also been the subject of chemical and thermal stability studies.^10,14,15^ Namely, nanometric CdS particles exhibit enhanced thermal resistance to the cubic-to-hexagonal phase transformation. The results of studies on the stability of CdS in aqueous solutions are also interesting. It was found that, despite the lack of resistance of CdS to corrosion and photocorrosion in aqueous electrolytes^16^ it can gain resistance when the size is in the nanoscale. Nanoparticles deposited on the surface of TiO_2_ gain stability and do not undergo photocorrosion reactions in aqueous electrolytes.^10,15^

At ambient temperature and pressure, CdS crystallizes in either hexagonal wurtzite (stable) or a cubic zinc blende (metastable) structure. However, the stability of CdS phases at the nanoscale is still under debate. Reports can be found supporting either higher stability of hexagonal^17^ or cubic phase^18^ as well as a mixture of these phases.^19^ This is significant because crucial properties of cadmium sulfide, such as electronic structure, band-gap energy, recombination of photogenerated charge carriers, and effective masses of electrons and holes,^20,21^ are sensitive to the crystallographic structure. Therefore, the structure has a strong impact on the photoinduced redox processes.^22^ Bao et al. reported the effects of the phase structure and phase composition of the CdS nanocrystals on the efficiency of photocatalytic H_2_ production.^23^ Muruganandham et al. reported the synthesis of microtowers and octahedral geometric CdS nanostructures and their photocatalytic H_2_-evolution activity.^24^ Lang et al. reported the influence of selected shapes of CdS nanocrystals on phase composition and photocatalytic hydrogen production activity.^1^ Shen et al. observed an increase in the photocatalytic activity of nanostructured CdS toward the degradation of methyl orange, which was accompanied by an increase in the proportion of the hexagonal phase.^25^

In this paper, we report CdS nanopowders with controlled phase composition and electron properties by Cd_1+*x*_S deviation from stoichiometry precipitated from water- and methanol-based precursors. Our aim was to use a simple, cost-effective, and environmentally friendly synthesis method. At the same time, the chosen method gives us control over the elemental composition. This allowed us to find a relationship between the nonstoichiometry, cadmium content, phase composition, and energy of the band gap.

## 2 Experimental Section

### 2.1 Materials

The following reactants were used in the procedure for the fabrication of nanometer CdS particles: Cd(NO_3_)_2_·4H_2_O (ACS reagent, ACROS), Na_2_S·9H_2_O (98%, Sigma-Aldrich), CH_3_OH (99.8%, Avantor Performance Materials Poland S.A.).

### 2.2 Preparation of CdS Powders

Cadmium nitrate(V) and sodium sulfide were dissolved separately in distilled water or methanol. The concentrations of the aqueous solutions of precursors were 0.001, 0.01, 0.1, or 0.2 M, while the concentrations of the methanol-based solutions were 0.01 or 0.001 M. The lack of methanol solutions with higher concentrations is due to the fact that the solubility of the precursors in this case is considerably lower than that in water. Regardless of the concentration and base of the precursors, they were combined in a 1:1 molar ratio. A yellow, orange, and orange precipitate of CdS was obtained. The precipitate was then rinsed with distilled water or methanol several times, filtered, and dried under reduced pressure in a vacuum dryer at 55 °C for 60 h. Subsequently, the dry CdS powder was ground in an agate mortar. Nanopowders formed in water-based solutions are denoted as CdS(w), while those formed in methanol-based solutions are denoted as CdS(m).

### 2.3 Experimental Methods

Transmission electron microscopy (TEM) images of CdS nanopowders were recorded on a JEOL-JEM1011 microscope. The particle size distribution was analyzed using ImageJ software.^26^ The chemical composition of the precipitate was investigated by energy-dispersive X-ray spectroscopy (EDX). Structural analysis was performed by X-ray diffraction (XRD) within the 2θ range of 20–80° using an X’Pert Pro (Panalytical) diffractometer with Cu Kα radiation. Phase identification was carried out with the X’PertHighScore Plus software and the PDF database. The content of phases was obtained by fitting the background and Gaussian–Lorentz profile to the experimental data in the EXPGUI software (a graphical user interface for GSAS). The parameters and volume of the unit cell as well as crystal size were calculated based on the Rietveld refinement of the XRD data with the use of the GSAS/EXPGUI software set.^27,28^ Simulated nanometric CdS diffraction patterns were calculated using GSAS software^27,28^ assuming various cubic-to-hexagonal CdS ratios. Peak broadening as a result of varying crystallite size was modeled under the assumption of a constant number of counts and the Gaussian shape of the peaks. Raman spectra were measured using a Jobin-Yvon HR800 spectrometer equipped with an Olympus confocal microscope (objective magnification of 100×) with a nitrogen-cooled CCD detector. Excitation was carried out using a laser beam with a wavelength (λ) of 532 nm and a diffraction grating equal to 1800 lines/mm. The optical and electronic properties of the samples were studied on the basis of the spectral dependence of the total reflectance (*R*_tot_) measured over a wide range of 220–2200 nm with a Jasco V-670 UV–vis–NIR double-beam spectrophotometer equipped with a 150 mm integrating sphere. The applied step was 0.5 nm, and the speed was 200 nm·min^–1^. The energy of the band gap (*E*_g_) was calculated from the Kubelka–Munk function.^29^

## 3 Results

Analysis of the chemical composition of CdS nanopowders performed with the use of EDX included powders obtained in water-based solutions. It was found that there was a clear relationship between the concentration of water-based precursors used in the precipitation process and the cadmium-to-sulfur ratio in the nanoparticles (Figure S1). The Cd/S ratio is equal to 1:0.980, 1:0.959, 1:0.910, and 1:0.699 for powders obtained in precursor solutions with concentrations of 0.001, 0.01, 0.1, and 0.2 mol·dm^–3^, respectively. Deviating from the equimolar composition was also recently reported by Devi et al.^30^ and Mohammed et al.^31^ for different methods of synthesis and concentration range.

The microstructure of the CdS nanopowders analyzed with TEM (Figure S2) revealed that precipitation from both water- and methanol-based solutions results in a round and uniform shape of the agglomerated nanoparticles. Analysis of the particle size distribution shows that in the case of CdS(w), the mean and median are equal to *x̃* = 7.1 nm and *x̅* = 7.0 nm, whereas mode *M*_O_ = 4 nm. On the other hand, for CdS(m), *x̃* = *x̅* = 5.5 nm and *M*_O_ = 5.0 nm. It shows that the distribution of the particle size of the powder precipitated from the methanol-based solution is narrower than that of the water-based one. The reason for this can be found in the fact that water and methanol differ greatly in their physical properties. Table S1 summarizes selected physical parameters of water and methanol, namely, density, surface tension, viscosity, boiling point, and dielectric constant. All of those values are much lower for methanol, which facilitates mass transport and mixing, reduces the degree of supersaturation, lowers the interfacial energy between the solution and the particles, results in weaker intermolecular interactions, makes nucleation more efficient, and, as a result, promotes the formation of smaller particles uniform in size.^10,32−34^

Analysis of Raman spectra (Figure S3) allows us to identify four optical vibrational Raman modes at approximately 300, 410, 600, and 900 cm^–1^. Three broad bands at 300, 600, and 900 cm^–1^ are assigned to the fundamental optical phonon mode (LO), the first overtone mode (2LO), and the second overtone (3LO) of CdS, respectively, and remain in agreement with the previously reported for CdS nanomaterials.^35^ An additional low-intensity band at approximately 410 cm^–1^ can be assigned to the vibrational mode 1LO + 2E_2_ and results from multiphonon scattering.^36−38^

It should also be noticed that the first-order LO Raman line is asymmetric compared to bulk CdS. The asymmetrical shape of the band is caused by the presence of two components: the low-frequency shoulder and the high-frequency shoulder. The first of them is attributed to surface optical (SO) phonons observed for nanostructures, and the second one is attributed to longitudinal optical (LO) phonons. The presence of both is characteristic of nanometric CdS with a particle size of a few nanometers.^36,39^

**Figure 1:**
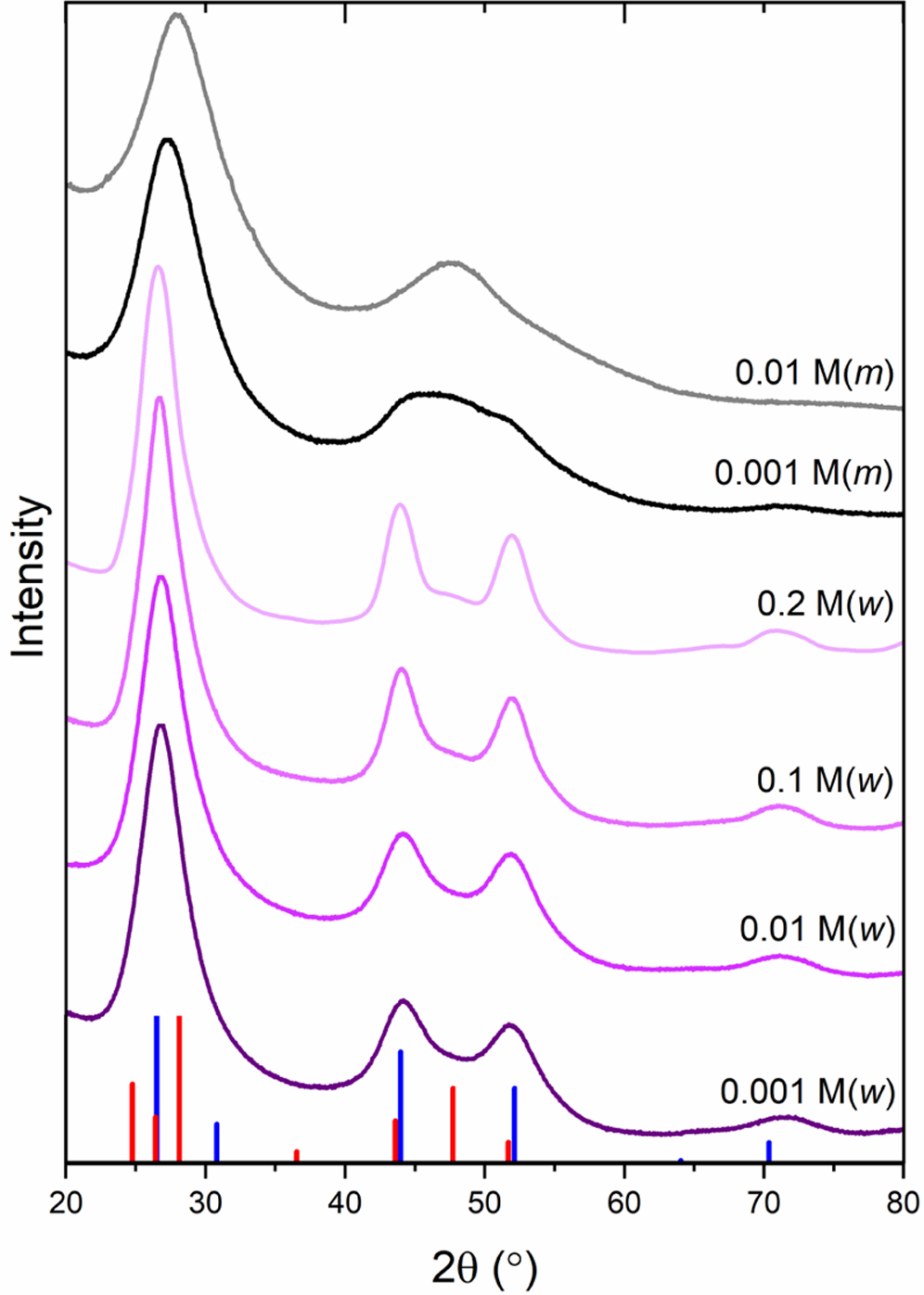
X-ray diffraction patterns of CdS nanopowders. The vertical markers correspond to the positions of the peaks of the cubic (blue) and hexagonal (red) phases.

XRD studies (Figure 1) revealed a distinct influence of precipitation conditions on the crystal structures of CdS nanopowders. The shape of the diffraction pattern is similar for the same solvent, irrespective of changes in concentration; however, it strongly depends on the type of solvent, indicating that surface effects play an important role. Analysis of the shape of the peaks revealed slight differences between the samples. Between 40 and 60°, a decrease in peak width and an increase in intensity are observed along with an increase in the concentration of the solvent. This could suggest an increase in crystallinity and an accompanying increase in crystallite size. However, the analysis of the size of CdS crystallites size shows that regardless of the initial concentration of the precursors, the size of the crystallites does not change and is close to 2.6 ± 0.1 nm. On the other hand, nanopowders crystallize as a mixture of cubic and hexagonal polymorphs and the contribution of the hexagonal phase grows from 49.2 to 60.3% with the concentration of the precursor. Therefore, the observed increase in the intensity in the 40–60° range can be attributed to the increasing contribution of the hexagonal phase.

For methanol-based precursors, the contribution of the hexagonal phase is greater than 70 wt % for 0.001 M and even greater than 90 wt % for 0.01 M. Those CdS nanopowders were characterized by a much smaller degree of crystallization and broader peaks. Detailed results of structural analysis are presented in Table 1.

**Table 1:** Structural Parameters of the CdS Nanopowders

	hexagonal CdS					cubic CdS		
	parameters of the unit cell							
precursor concentration (M)	*a* (nm)	*c* (nm)	volume of unit cell (nm^3^)	content (%)	crystal size (nm)	parameter of unit cell *a* = *c* (nm)	volume of unit cell (nm^3^)	crystal size (nm)
	Water-based							
0.001	0.4141(4)	0.653(2)	0.097(1)	49.2(1)	2.7(2)	0.5805(6)	0.1956(1)	2.5(2)
0.01	0.4146(4)	0.656(2)	0.098(1)	52.1(1)	2.6(2)	0.5810(6)	0.1961(2)	2.5(2)
0.1	0.4148(4)	0.675(1)	0.101(1)	58.8(1)	2.6(2)	0.5849(5)	0.2000(9)	2.5(2)
0.2	0.4150(1)	0.681(1)	0.102(1)	60.3(1)	2.7(2)	0.5855(1)	0.2007(1)	2.5(2)
	Methanol-based							
0.001	-	-	-	>70	2.7(2)	-	-	2.5(2)
0.01	-	-	-	>90	2.7(2)	-	-	2.5(2)

The parameters of the unit cell of the hexagonal CdS polymorph are equal to *a* = 0.41348 nm and *c* = 0.6749 nm, while for the cubic phase, *a* = *c* = 0.5818 nm. Comparison of the data from the literature and our experimental values revealed some differences that might be attributed to imperfections in the crystal lattice associated with the nanometric size of the particles. When the crystallite size is of the order of several unit cells, the contribution of surface atoms radically increases, and the unit cells become distorted. An increase in the concentration of water-based precursors is accompanied by a slight increase in the parameters of the unit cell. Unit cell parameters of CdS(m) nanoparticles are not presented because of the high error of determination.

**Figure 2:**
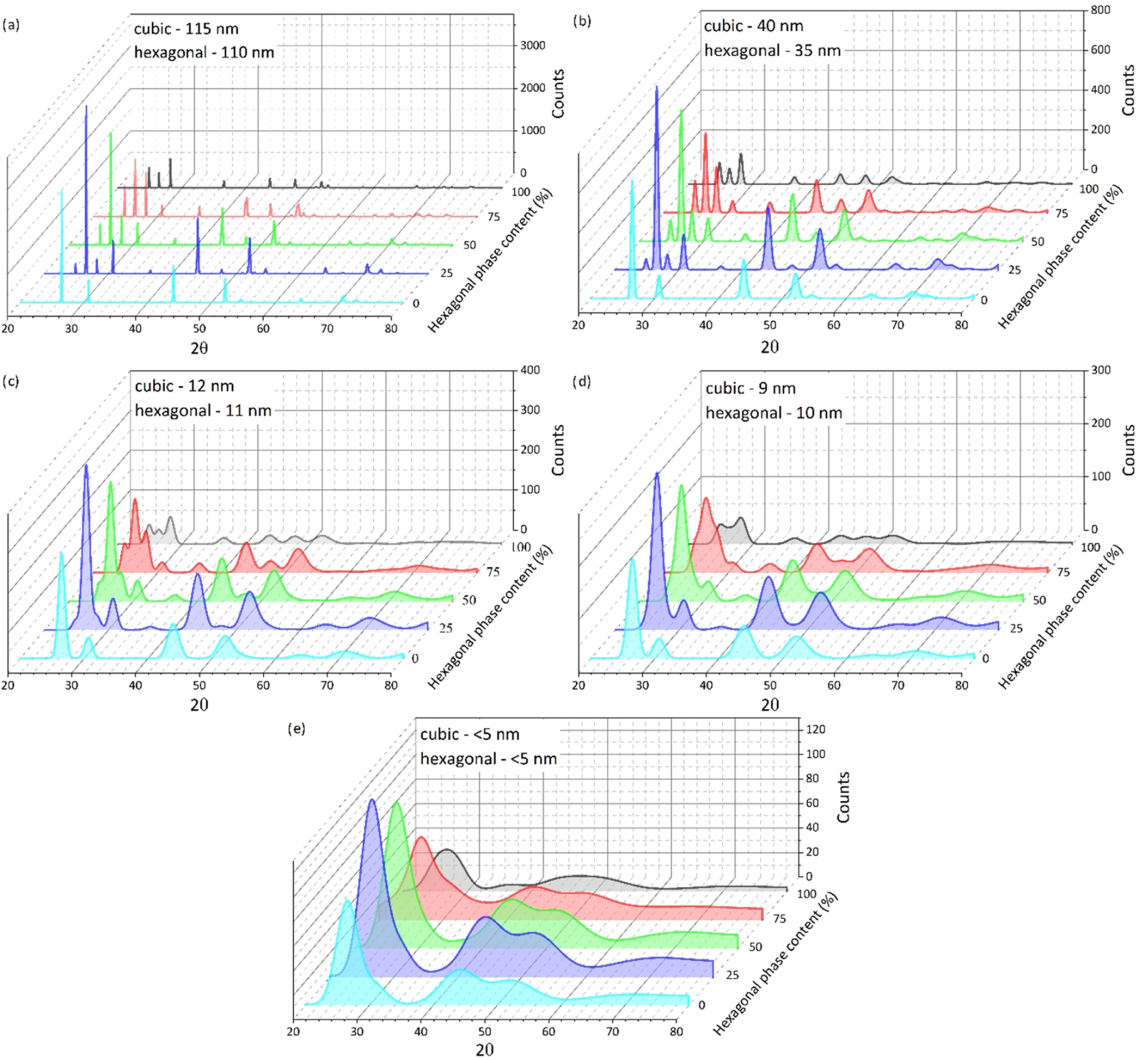
(a–e) Simulated XRD patterns of CdS nanopowders characterized by different sizes of crystallites and different contributions of the hexagonal phase.

However, it should be noted that XRD analysis of small crystallites poses some problems. First, differentiating the CdS hexagonal structure from the cubic structure can be difficult. The reason for this is that the positions of the main peak (intensity of 100%) of both phases, (002) hexagonal and (111) cubic, coincide within 1%. This problem does not apply to crystallized materials with relatively large crystallites. In such a case, the peaks are well separated. For better visualization, the results of the simulation of the diffraction patterns of single- or two-phase nanometric CdS are presented in Figure 2. When the size of CdS crystallites is large enough, there is a clear difference in the shape of the diffraction pattern of the nanopowders, differing in phase content. There is, however, a certain limit below which the shapes of the peaks become increasingly similar. The border appears to be ∼10 nm (Figure 2d); up to 50% of the hexagonal polymorph differences are subtle, and below 5 nm only the hexagonal single-phase material is distinctly different from the mixture of phases.

**Figure 3:**
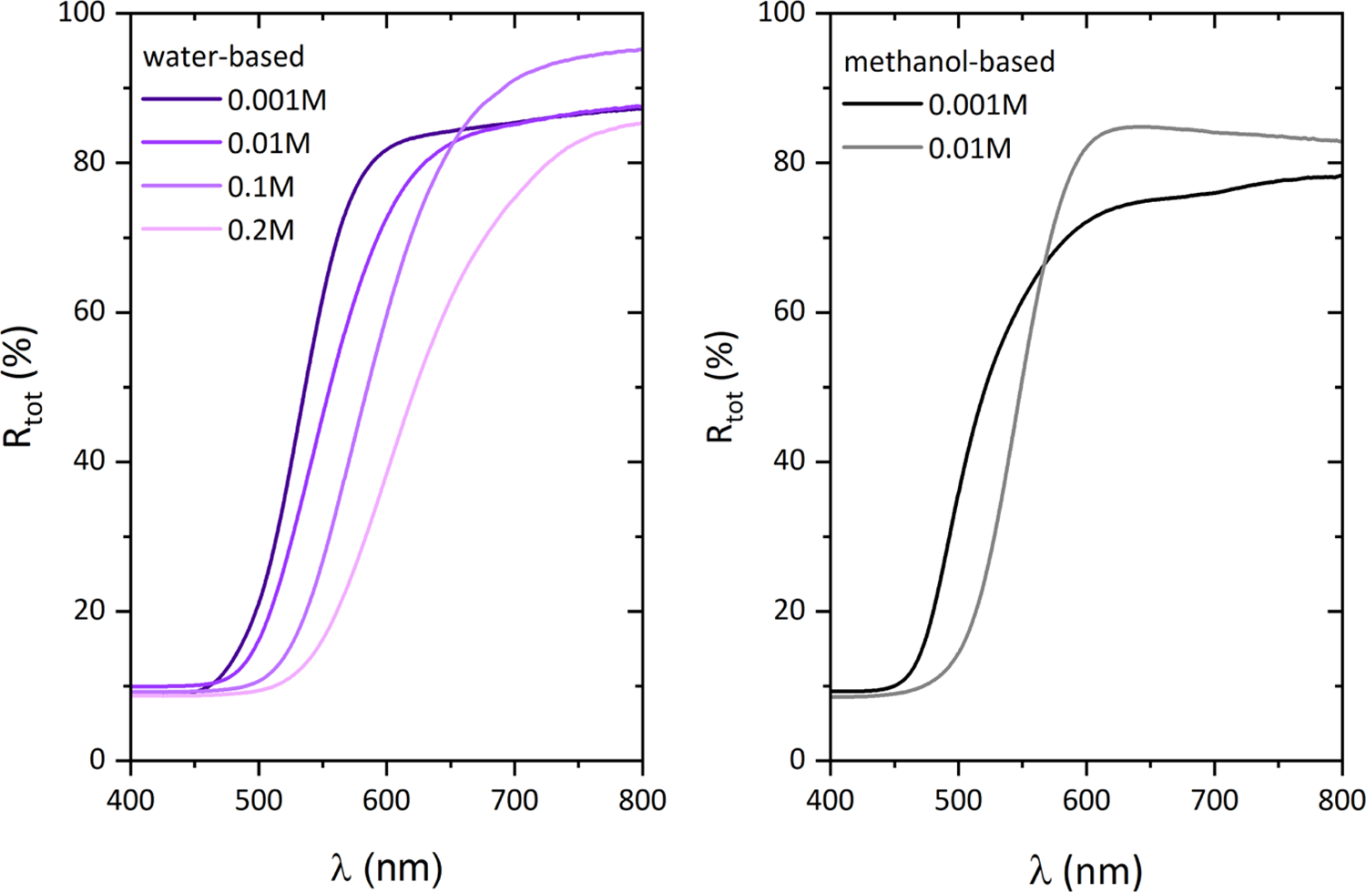
Spectral dependence of the total reflectance of CdS nanopowders precipitated from (left) water- and (right) methanol-based precursor solutions.

The optical and electronic properties of the CdS nanopowders were evaluated based on the spectral dependences of the total reflectance and are presented in Figure 3. Between 350 and 700 nm, a sudden change in reflectance is observed, which is a fundamental absorption edge. It arises directly from the light-induced electron transfer from the valence to the conduction band. In the case of both water- and methanol-based precursor solutions, along with an increase of the concentration of the solution in the precipitation process, a systematic shift of the fundamental absorption edge toward longer wavelengths occurs.

The band-gap energy was calculated based on the spectral dependence of reflectance (Figure 3) using the Kubelka–Munk function,^29^ and the obtained data are gathered in Table 2. The method of determining the energy of the band gap by the Kubelka–Munk approach is shown in Figure S4. Analysis revealed that *E*_g_ increases along with the decreasing concentration of precursor. This could be a consequence of the quantum size effect, which in the case of CdS is observed for particles less than 60 nm in size.^40^ However, in our case, no change in the size of the nanometric particles is observed.

**Table 2:** Band gap, Absorption Onset, Band-Gap Shift, Lattice Parameters, and Crystal Size of CdS Nanopowders. Band-Gap Shift, Δ*E*_g_, Was Calculated with respect to That of Bulk CdS: *E*_g_ = 2.42 eV

		Δ*E*_g_	
precursor concentration (M)	band-gap energy (eV)	(eV)	%
Water-based			
0.001(w)	2.49(5)	+0.07	+2.9
0.01(w)	2.41(5)	–0.01	–0.4
0.1(w)	2.27(5)	–0.15	–6.2
0.2(w)	2.21(5)	–0.21	–8.7
Methanol-based			
0.001(m)	2.56(5)	+0.14	+5.8
0.01(m)	2.40(5)	–0.02	–0.8

## 4 Discussion

The concentration at which nucleation begins is defined by the solubility product (*K*_s_). For CdS, *K*_s_ = 2.51 × 10^–26^,^41^ which means that only 2.29 × 10^–11^ g (1.59 × 10^–13^ mole) of CdS can be dissolved in 1 dm^3^ of water to obtain a saturated solution. In practice, this means that mixing the Cd^2+^ and S^2–^ solutions will result in the precipitation of CdS (however, the rate of precipitation will depend on the kinetics of the reaction). Nucleation, the first step in crystallization, can be either spontaneous or induced by the vibration of particles; yet, the kinetics of nucleation is crucial to the resulting size of crystals.

A lower concentration creates conditions that lead to limited growth of the forming nuclei compared with a higher concentrated solution. As a result, in solutions of lower concentration, nuclei crystallize into smaller crystals and in solutions of higher concentration, in larger crystals.^42,43^ In the case of cadmium sulfide, this is confirmed by numerous papers showing the effect of precursor concentration on crystallite size.^44−47^ It seems particularly interesting that, in the case of our nanomaterials, this relationship is not observed, which will be the subject of further research. Analysis of the diffraction patterns of these crystals will result in a difference in full width at half-maximum (fwhm) and a higher degree of crystallization. Such conclusions can be reached in Figure 1 showing the nanopowders obtained from the aqueous solutions. However, these conclusions are incorrect. Although an increase in precursor concentration is accompanied by a decrease in fwhm and is expressed by a sharper peak profile, this is only apparent and is due to a change in the ratio of polymorphic varieties of regular and hexagonal CdS. Therefore, the influence of the solvent should also be regarded.

Replacing water with methanol leads to major changes in the diffraction pattern that manifest themselves, among others, by higher fwhm. Comparison of the physical properties of both solvents, methanol and water, shows some basic differences regarding viscosity, density, and surface tension. First, the viscosity is higher for water, i.e., 8.9 × 10^–4^ Pa·s, whereas for methanol, it is 5.44 × 10^–4^ Pa·s.^48^ The influence of viscosity on the morphology of the deposited particles was already observed in the case of electrophoretic deposited TiO_2_.^49^ The use of a medium with a higher viscosity led to a smaller number of deposited particles and their uniform distribution on the substrate surface. Moreover, the size distribution of the deposited particles was much wider when the viscosity was low. Next, the density of methanol is lower than that of water, 0.7914 versus 0.9970 g·cm^–3^,^48^ which also favors nucleation of numerous nuclei much easier than in the water environment.

**Figure 4:**
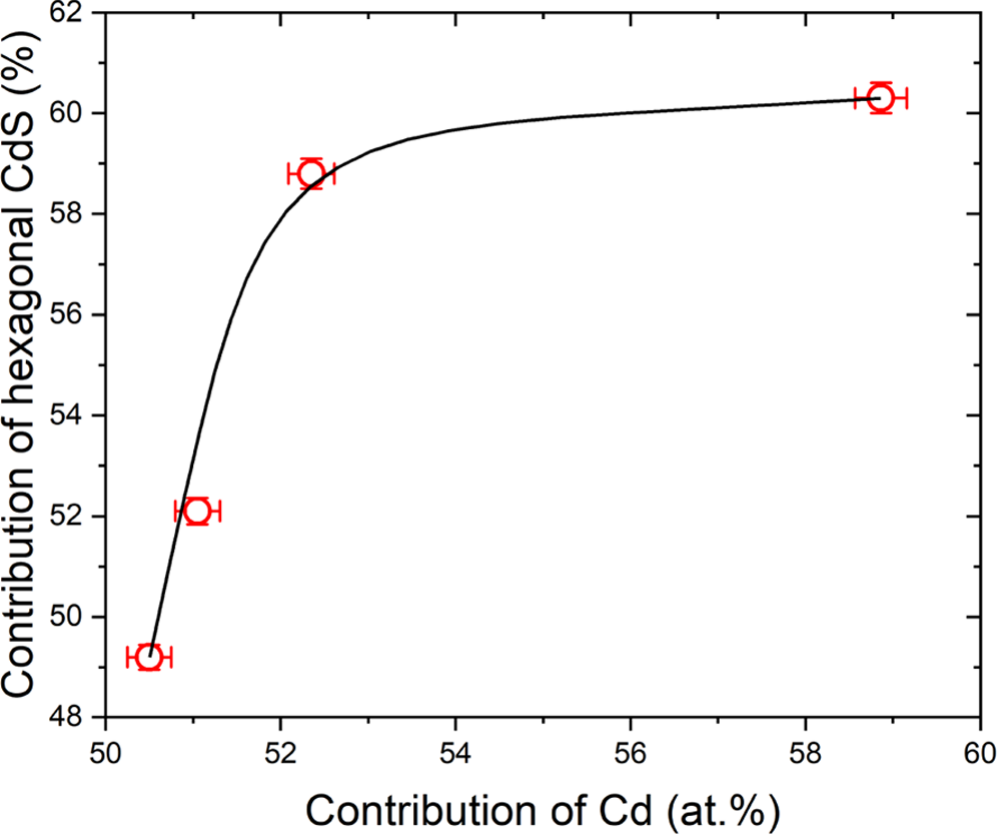
Effect of chemical composition on the contribution of the CdS hexagonal phase.

Another interesting observation is the relationship between the elemental compositions of the nanopowders and their phase compositions (Figure 4). An increase in precursor concentration during precipitation is accompanied by an increase of the cadmium-to-sulfur ratio. In stoichiometric cadmium sulfide, the Cd/S ratio should be equal to 1. However, sulfides are known for their susceptibility to having their composition deviate over a wide range from a stoichiometric one. Nonstoichiometry in cadmium sulfide can be manifested by the presence of cadmium vacancies (V_Cd_), cadmium interstitial atoms (Cd_i_), sulfur vacancies (V_S_), sulfur interstitial atoms (S_i_), cadmium in the sulfur position (Cd_S_), sulfur in the position of cadmium (S_Cd_), or stacking faults.^50^ In this particular case, when Cd/S > 1, nonstoichiometry could result from an actual or apparent deficiency of cadmium or excess of sulfur. According to Lozada-Morales et al., these defects may contribute to the phase transition between cubic and hexagonal structures.^51^Figure 4 shows that a change of 8.3 percentage points of Cd contribution (an increase from 50.6 to 58.9 atom %) leads to an increase in the contribution of the hexagonal phase by more than one-fifth (from 49.0 to 60.5%). This indicates that the consequence of the deviation from stoichiometry is CdS in the hexagonal polymorph. The outcome of the predominant contribution of Cd compared to that in the stoichiometric CdS is the preferred crystallization of the hexagonal phase, which is more stable under such conditions. Similar conclusions were reached by Datta et al. based on computations of density functional theory.^21^ What they concluded on the basis of their calculations was preferential growth of the hexagonal phase when Cd^2+^ ions predominated on the CdS surface and preferential growth of the regular phase when S^2–^ ions predominated on the surface. This suggests that interstitial cadmium Cd_i_ or sulfur vacancy V_S_ is responsible for the preferred hexagonal phase growth. Theoretical expectations were also experimentally confirmed by Nanda et al.^52^ who found that as the cadmium-to-sulfur ratio increases, the amount of hexagonal phase increases.

When looking at the bulk *E*_g_ and comparing it with CdS nanopowders prepared from the solution of the lowest concentration (0.001 M), it can be seen that there is a strong blueshift of the value. This could be attributed to the quantum size effect. When the concentration increases 10-fold, the band gap is shifted to the expected value of bulk CdS, which is 2.42 eV. However, *E*_g_ continues to shift to the red as the concentration increases and becomes more pronounced with a final energy of about 2.21 eV for a concentration equal to 0.2 M. It should also be noted that the red shift is accompanied by the change of the CdS nanopowder from light yellow to orangish along with the increasing concentration during precipitation.

The modification of the value of *E*_g_ due to the change of the size of particles can be achieved in different ways by changing the time (2.31 eV^53^) or temperature (2.20 eV^54^) of the deposition process, by annealing (2.28 eV^55^), or by changing the thickness of the deposited layer (2.03–2.35 eV^10,56−58^). Although the red shift of the CdS band gap is often reported in the literature, there are only a few papers that describe strong red shifts of the band gap giving an explanation of their probable origin. The predominant number of articles refer to CdS deposited by the SILAR method on the surface of wide-band semiconductors such as TiO_2_ or ZnO.^59−62^ According to those papers, the red shifts of *E*_g_ appear to be due to a ‘true shift’ of the spectral dependence. The term “true shift” is understood to be a shift of the fundamental absorption edge caused by modifying the position of the upper edge of the valence band and/or the lower edge of the conduction band or else the presence of localized defect levels originating from nonstoichiometry, doping, or surface state. This stands in opposition to the situation in which the shift is actually a blurring of the fundamental absorption edge. Such an effect is observed when there is significant absorption of sub-band-gap energy photons and results from heavily disordered structures due to strong doping, large deviation from stoichiometric composition, or amorphousness.^63,64^ However, there are some studies that identify the red shift phenomenon that appears in the optical response of CdS as an exceptionally long absorption tail that stretches unusually far into the red region. Rabinovich and Hodes observed the red shift for CdS deposited on TiO_2_ using the SILAR method.^57^ As the amount of CdS particles increased (along with the number of SILAR cycles), a greater red shift was observed. It was interpreted to be due to the large optical thickness of high-surface-area nanoporous films and localized states near the band gap in CdS, which results from a high degree of structural disorder.^57^ Another explanation was presented by Tong et al. who calculated *E*_g_ to be equal to 2.18 eV.^65^ Their studies of chemical composition revealed that there was a nonstoichiometry in CdS related to excess cadmium or sulfur deficiency with the cadmium-to-sulfur ratio Cd/S = 1.17 (58.5% of Cd). On the basis of both experimental results and theoretical calculations, it was suggested that hybridization of Cd levels might occur in either Cd-rich or S-deficient CdS nanomaterials, and as a result, new energy levels could be created inside the forbidden energy region.^65^ Zhou et al. also found nonstoichiometry in CdS with Cd/S = 1.05 (52.5% of Cd) accompanied by *E*_g_ = 2.09 eV,^56^ and they have acceded to the earlier interpretation recalled by Tong et al. However, Zelaya-Angel et al. attributed an increase in Cd content or S deficiency, which was accompanied by an increase in the energy of the band gap, to the associated increasing contribution of the hexagonal phase.^66^

Analysis of our XRD and optical data shows that there is an explicit dependence between the contribution of the hexagonal phase and the band-gap energy (Figure S4). A linear decrease in *E*_g_ is observed along with an increase in the content of hexagonal CdS. Similar observations were made by Li et al. which they attributed to the difference in the band-gap energy between the cubic and hexagonal CdS polymorph.^67^ However, there is no evidence in the literature that supports this assumption. In addition, Mohammed et al.^31^ and Oluyamo et al.^68^ also found that as the Cd/S ratio increases, the band-gap energy decreases.

**Figure 5:**
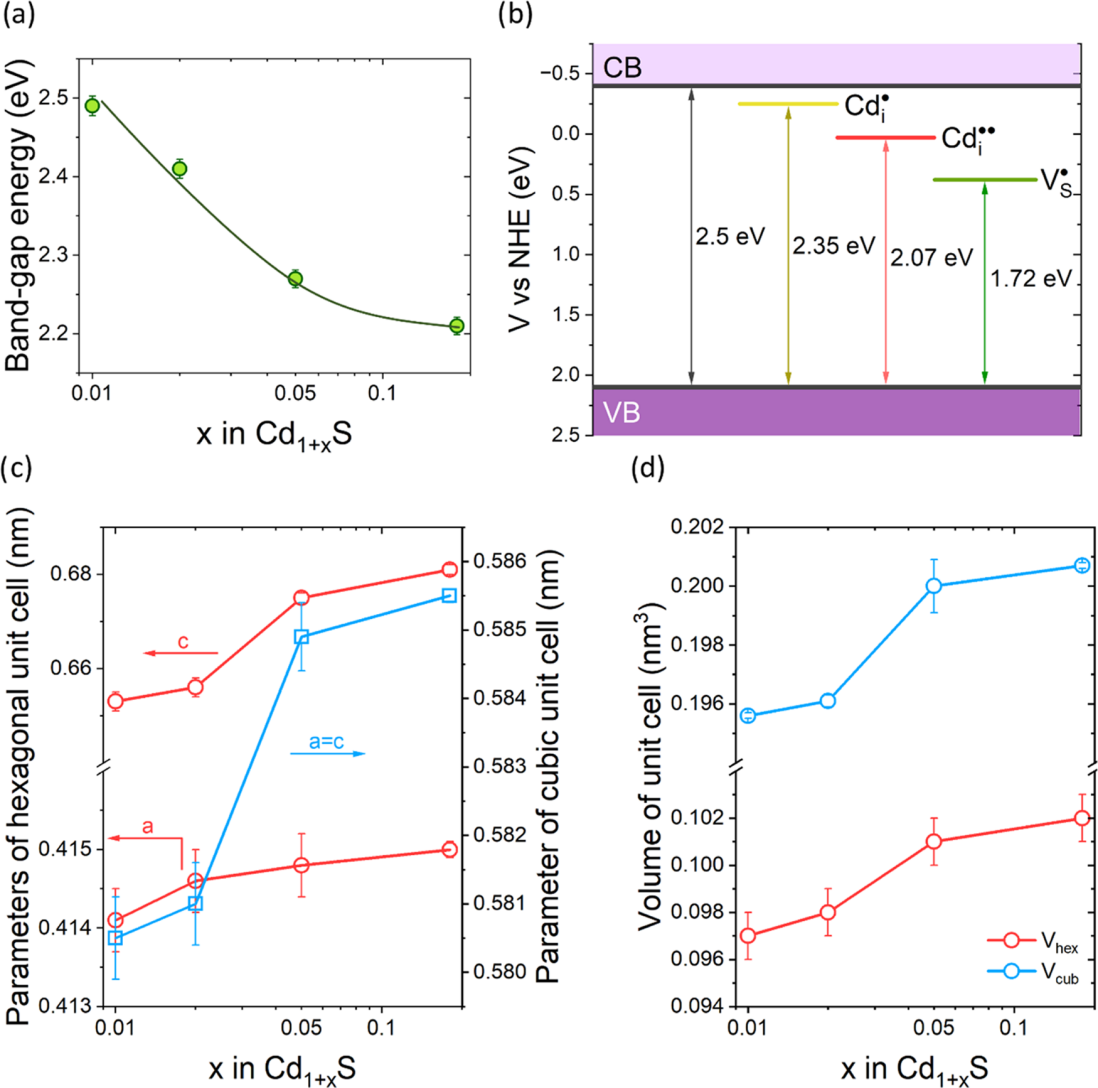
(a) Effect of the contribution of cadmium on the band-gap energy. (b) Diagram representing the position of selected levels of intrinsic point defects located in the forbidden band of CdS (based on ref (71)). Dependence of (c) unit cell parameters and (d) volume of unit cell on CdS nonstoichiometry.

The reason for the decrease in CdS band-gap energy accompanying an increase in the Cd/S can also be found elsewhere. The reduction in particle size and the transition from the micro- to the nanometric scale result in significant increases in the ratio of the surface area to the volume of the particle. Consequently, there are a particularly large number of surface atoms that have different properties than atoms within a particle. The bonds of the surface atoms are nonsaturated because the surface lacks the neighboring atoms to which bonds are formed inside the particle.^69^ This leads to tension. Therefore, increasing the development of the particles’ specific surface area causes the crystal structure of cadmium sulfide to be imperfect.^70^ As mentioned above, excess cadmium relative to sulfur can be caused by interstitial cadmium or sulfur vacancies. However, according to Varley and Lordi, V_S_, which form deep donor levels, are not significant in n-type semiconductors due to the low equilibrium concentration of these defects.^71^ The chemical formula of such a cadmium sulfide can be therefore expressed as Cd_1+x_S. Interstitial cadmium ions will be dominant, forming shallow donor levels in the band gap below the bottom edge of the conduction band. Figure 5b shows a diagram illustrating the position of the shallow Cd_i_ and deep V_S_ donor levels in the forbidden band of cadmium sulfide. Defect formation reactions toward the actual cadmium excess can be expressed as
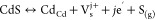
1
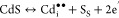
2

As the cadmium content in nonstoichiometric Cd_1+*x*_S increases, the concentration of interstitial singly Cd_i_^•^ and doubly Cd_i_^••^ ionized cadmium increases. As a consequence, this causes a partial deformation of the crystal lattice. This is confirmed in our case by XRD data, analysis of which is presented in Figure 5c,d. Growth of the unit cell parameters of both polymorphic varieties of CdS is observed in the materials studied. The size of the unit cell increases with increasing deviation from the stoichiometric composition; in the hexagonal polymorph, the parameter *a* grows by 0.2% and the parameter *c* by 4.3%, while in the cubic structure, *a* = *c* increases by 0.86%. This results in an increase in the unit cell volume equal to 5.2 and 2.6%, respectively. The consequence of the presence of defect levels in the forbidden gap is a decrease in the energy of the CdS band gap. As can be seen in Figure 5a, an increase in x in Cd_1+*x*_S results in a decrease in *E*_g_ from 2.49 to 2.21 eV.

The presence of point defects, such as interstitial cadmium, modifies the band structure of the semiconductor. This, in turn, strongly affects electron transfer in processes based on redox reactions occurring at the semiconductor–electrolyte interface. Additional levels in the forbidden energy band of Cd_1+*x*_S originating from interstitial cadmium mediate the electron transfer at the interface. It is also significant that cadmium sulfide that exhibits a deviation from the stoichiometric composition also shows additional absorption of energy quanta from the visible range with energy lower than for stoichiometric CdS. Therefore, modification of the electron structure of the semiconductor–electrolyte interface, combined with extended absorption of radiation by longer wavelengths, can be predicted to lead to enhanced efficiency of redox reactions in photochemical processes. Confirmation of this assumption can be found in the experimental work carried out by Arora et al. in which it was shown that the presence of interstitial cadmium was associated with enhanced photocatalytic activity of Cd_1+*x*_S toward hydrogen production from water.^72^

## Conclusions

It was found that increasing the concentration of precursor solutions composed of Cd(NO_3_)_2_ and Na_2_S in both water- and methanol-based solutions during the precipitation process results in an increase in the cadmium-to-sulfide ratio of the resulting CdS nanopowders. The resulting nonstoichiometry causes the preferred crystallization of the hexagonal phase. This is due to the presence of interstitial cadmium, which leads to an increase in the lattice parameters and unit cell volume. The interstitial cadmium also forms shallow donor levels in the forbidden energy band, which decreases the energy of the band-gap energy and broadens the light absorption range. This can increase the efficiency of the redox reactions in photochemical processes.

## Supporting Information Available

Dependence of Cd^2+^ contribution on the concentration of Cd^2+^ and S^2–^ precursor, TEM images, physical properties of water and methanol, Raman spectra, method of determining the band-gap energy from the Kubelka–Munk function, and effect of the hexagonal phase contribution on band-gap energy (PDF)
