# Supplementary material for: Rational Design of the Electronic Structure of CdS Nanopowders
Source: Inorg Chem. 2023 Jun 29;62(28):10955–64. doi: 10.1021/acs.inorgchem.3c00935 (PMC10354744; doi:10.1021/acs.inorgchem.3c00935)
Supplement: Supplementary file 1 — ic3c00935_si_001.pdf [file ic3c00935_si_001.pdf]

Electronic Supporting Information

On

Rational design of electronic structure of CdS nanopowders

W. Zajac[1], A. Rozycka[2], A. Trenczek-Zajac[2*]

[1]Faculty of Energy and Fuels, AGH University of Science and Technology, al. Mickiewicza 30, 30-059, Krakow, Poland

[2]Faculty of Materials Science and Ceramics, AGH University of Science and Technology, al. Mickiewicza 30, Krakow, Poland

*corresponding author: anita.trenczek-zajac@agh.edu.pl

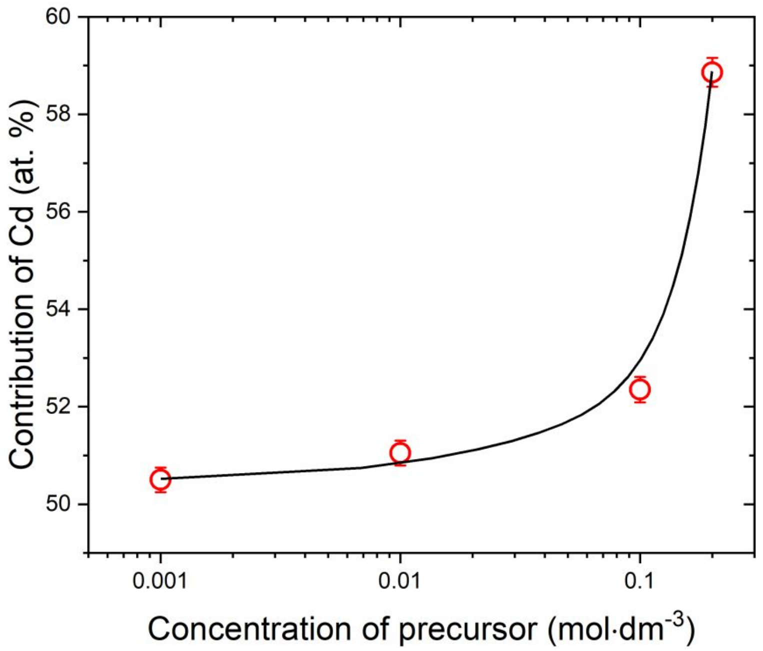

Figure S1. Dependence of contribution of cadmium on the concentration of Cd^{2+} and S^{2-} in water-based precursor solutions in precipitation process.

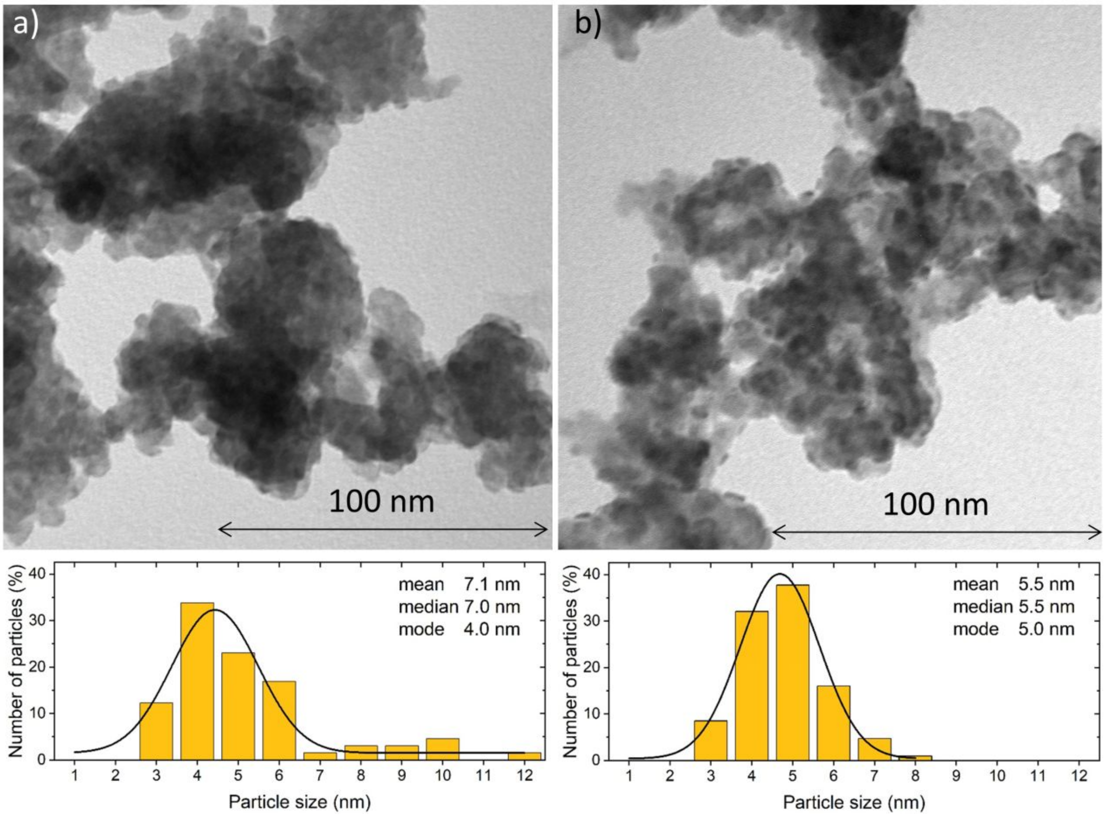

Figure S2. TEM images and particle size distribution of CdS nanopowder prepared from (a) 0.01M water- and (b) 0.01M methanol-based precursors.

Table S1. Selected physical properties of water and methanol [S1].

	Water	Methanol
Density at 25°C (g·cm^{-3})	0.9970	0.7914
Surface tension (g·s^{-2})	71.99	22.07
Viscosity (Pa·s)	$8.9·10^{-4}$	$5.44·10^{-4}$
Dielectric constant	80.1	33.0
Boiling point (^{o}C)	100	64.6

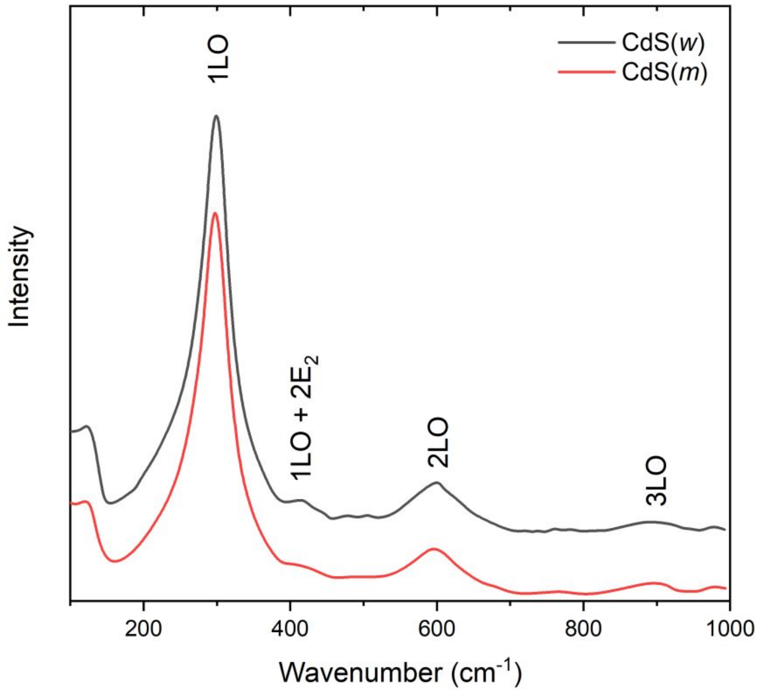

Figure S3. Raman spectra of CdS prepared from water- and methanol-based solutions.

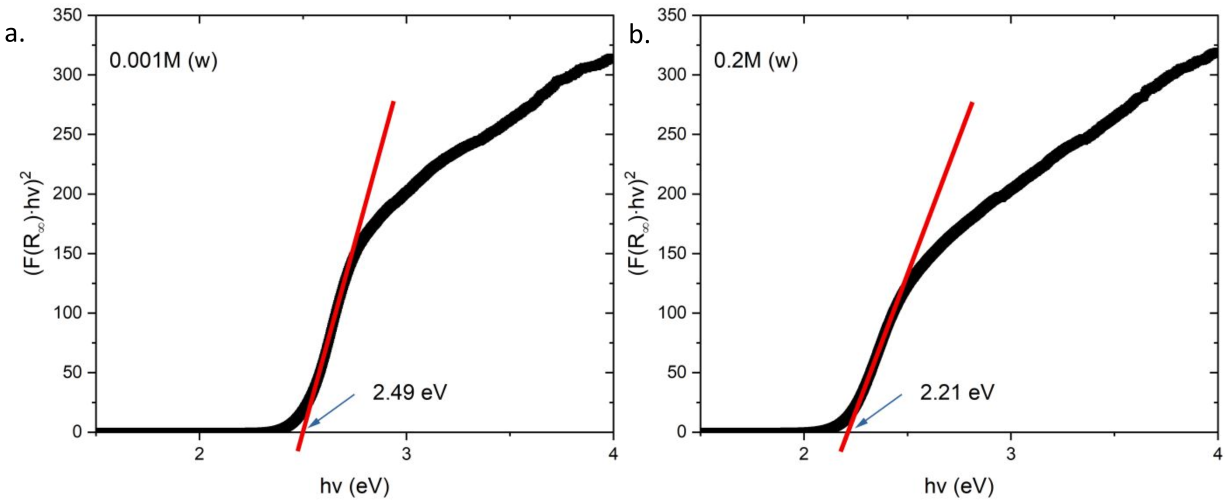

Figure S4. Method of determining the band gap-energy from the Kubelka-Munk function for CdS nanopowders: a. 0.001M (w) and b. 0.2M (w).

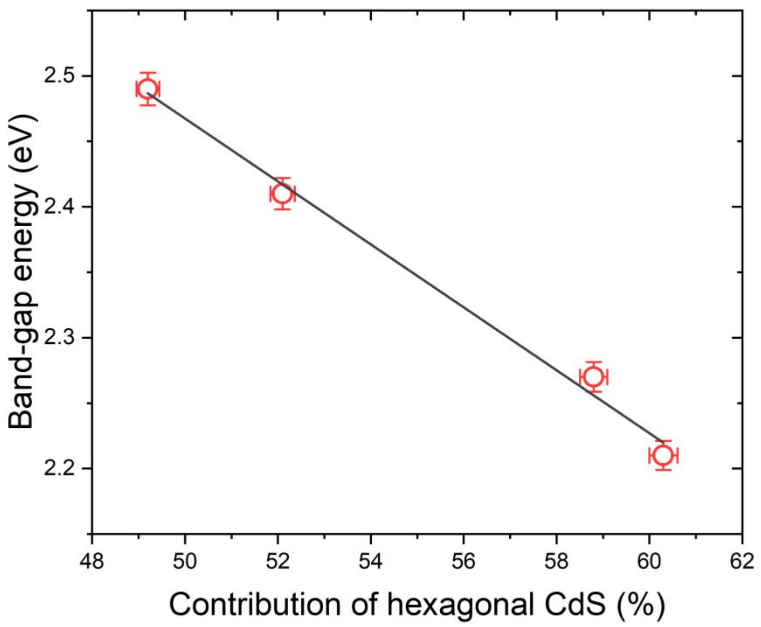

Figure S5. Effect of the hexagonal phase contribution on band-gap energy.

References

[S1] *CRC Handbook of chemistry and physics,* Edited by: J.R. Rumble, 2022
